# Inhibition of angiogenesis- and inflammation-inducing factors in human colon cancer cells *in vitro *and *in ovo *by free and nanoparticle-encapsulated redox dye, DCPIP

**DOI:** 10.1186/1477-3155-8-17

**Published:** 2010-07-15

**Authors:** Fadee G Mondalek, Sivapriya Ponnurangam, Janita Govind, Courtney Houchen, Shrikant Anant, Panayotis Pantazis, Rama P Ramanujam

**Affiliations:** 1 Swaasth, Inc., 800 Research Parkway Suite 350, Oklahoma City, OK 73104 - USA; 2 University of Oklahoma Health Sciences Center, College of Medicine, Oklahoma City, OK 73104 - USA; 3 University of Oklahoma Health Sciences Center, Department of Cell Biology, Oklahoma City, OK 73126 - USA; 4 ADNA, Inc., Research Parkway Suite 350, Oklahoma City, OK 73104 - USA

## Abstract

**Background:**

The redox dye, DCPIP, has recently shown to exhibit anti-melanoma activity *in vitro *and *in vivo*. On the other hand, there is increasing evidence that synthetic nanoparticles can serve as highly efficient carriers of drugs and vaccines for treatment of various diseases. These nanoparticles have shown to serve as potent tools that can increase the bioavailability of the drug/vaccine by facilitating absorption or conferring sustained and improved release. Here, we describe results on the effects of free- and nanoparticle-enclosed DCPIP as anti-angiogenesis and anti-inflammation agents in a human colon cancer HCT116 cell line *in vitro*, and in induced angiogenesis *in ovo*.

**Results:**

The studies described in this report indicate that (a) DCPIP inhibits proliferation of HCT116 cells *in vitro*; (b) DCPIP can selectively downregulate expression of the pro-angiogenesis growth factor, VEGF; (c) DCPIP inhibits activation of the transcriptional nuclear factor, NF-κB; (d) DCPIP can attenuate or completely inhibit VEGF-induced angiogenesis in the chick chorioallantoic membrane; (e) DCPIP at concentrations higher than 6 μg/ml induces apoptosis in HCT116 cells as confirmed by detection of caspase-3 and PARP degradation; and (f) DCPIP encapsulated in nanoparticles is equally or more effective than free DCPIP in exhibiting the aforementioned properties (a-e) in addition to reducing the expression of COX-2, and pro-inflammatory proteins IL-6 and IL-8.

**Conclusions:**

We propose that, DCPIP may serve as a potent tool to prevent or disrupt the processes of cell proliferation, tissue angiogenesis and inflammation by directly or indirectly targeting expression of specific cellular factors. We also propose that the activities of DCPIP may be long-lasting and/or enhanced if it is delivered enclosed in specific nanoparticles.

## Background

It is well established that vascular endothelial growth factor (VEGF) plays a prominent role in the induction of physiological or pathophysiological processes of angiogenesis, vasculogenesis, arteriogenesis, and lymphangiogenesis collectively termed as vascularization [1-5]. However, although the evidence existing in the literature supports the idea that VEGF is a positive regulator of tumor growth, recently published reports indicate that VEGF also acts as a negative regulator of tumor growth [6,7]. In general, VEGF promotes angiogenesis by induction of the enzymes, cyclooxygenase-2 (COX-2) and nitric oxide synthase (iNOS), and overexpression of VEGF and COX-2 in cancer tissues has been reported to be associated with poor prognosis in patients with cancers [8-10]. COX-2 is an inducible enzyme produced by many cell types in response to multiple stimuli. Recently, COX-2 over-expression has been detected in several types of human cancers such as colon, breast, prostate, lung, pancreas and leukemias and appears to control many cellular processes [10]. Because of their roles in angiogenesis, carcinogenesis, and apoptosis, VEGF and COX-2 are excellent targets for developing new drugs with selectivity for prevention and/or treatment of human cancers.

Cytokine-mediated immunity plays a crucial role in angiogenesis, organogenesis, and the pathogenesis of various diseases including tumor development, growth and metastasis, atherosclerosis, sepsis, and rheumatoid arthritis. Major players in these processes are the pro-inflammatory cytokines, interleukin (IL)-6 and IL-8, which may or may not be produced following induction by VEGF depending on the tissue [11-14]. IL-6 has a wide range of biological activities including regulation of immune response, support of hematopoiesis, generation of acute-phase reactions and induction of inflammation and oncogenesis [11,15]. Importantly, overproduction of IL-6 from synovial cells is critically involved in the pathogenesis of rheumatoid arthritis, a chronic, debilitating disease in which articular inflammation and joint destruction are accompanied by systemic manifestations including anaemia, fatigue and osteoporosis [16-18]. In this context, IL-8 has been involved in the expression of VEGF in endothelial cells [19], induction of angiogenesis [20], tumor angiogenesis in gastric cancer [13], and acquisition of chemotherapeutic resistance in androgen-independent proliferation of prostate cancer cells [14]. Finally, there is extensive experimental and clinical evidence to indicate that the nuclear transcription factor-κB (NF-κB) is activated during, and therefore links, the processes of angiogenesis, inflammation and carcinogenesis [21-24].

The redox dye, 2,6-dichlorophenolindophenol (DCPIP), is a cell membrane-permeable oxidant widely used as a specific standard substrate for the colorimetric determination of cellular NAD(P)H:quinone oxidoreductase and as an oxidizing reactant [25,26]. DCPIP can be synthesized 99.5% pure as a dark green-black powder, is stable, odorless and freely soluble in water, and exhibits drug-like properties that include chemical stability, systemic deliverability, membrane permeability, and low systemic toxicity established in mice [27].

It has been long established that cell death by "classical" apoptosis initiated by caspase-8, i.e. death receptor-dependent apoptotic pathway, or caspase-9, i.e. mitochondrion-dependent pathway, results in activation of caspase-3, which in turn targets and degrades specific and vital cellular proteins, including the enzyme poly(ADP-ribose)polymerase (PARP), and apoptotic death of the cells [28,29]. Further, there have been reports on the existence of caspase-independent mechanisms of cell death executed by other proteases, thus leading to variant forms that may display some or no characteristics of the "classical" apoptosis pathways [30-32]. Finally, pertinent to apoptotic events is the report that following exposure of human melanoma cells to DCPIP in vitro results in activation, i.e. specific degradation, of procaspace-3 followed by apoptotic cell death [27]. However, there is no report on induction of apoptosis in human cells treated with DCPIP encapsulated in nanoparticles.

The use of poly(lactic-co-glycolic) acid nanoparticles (PLGA NPs) has emerged as a powerful potential methodology for carrying small and large molecules of therapeutic importance as well as scaffolds for tissue engineering applications. This utility derives primarily from: (a) physiological compatibility of PLGA and its monomers, polyglycolic acid (PGA) and polylactic acid (PLA), all of which have been established to be safe for human use for more than 30 years in various biomedical applications including drug delivery systems; (b) commercial availability of a variety of PLGA formulations for control over the rate and duration of molecules released for optimal physiological response [33,34]; (c) biodegradability of PLGA materials, which provides for sustained release of the encapsulated molecules under physiologic conditions while degrading to nontoxic, low-molecular weight products that are readily eliminated [35]; and (d) control over its manufacturing into nanoscale particles (< 500 nm) for potential evasion of the immune phagocytic system or fabrication into microparticles on the length scale of cells for targeted delivery of drugs or as antigen-presenting systems [36]. This unique combination of properties coupled with flexibility over fabrication has led to interest in modifying the PLGA surface for specific attachment to cells or organs in the body [37,38] for drug delivery and tissue engineering applications.

In this report, we demonstrate that (a) DCPIP enclosed in PLGA nanoparticles (DCPIP NPs) exhibits higher anti-proliferative activity than free DCPIP in cultured cancer HCT116 cells; (b) DCPIP NPs is similarly or more potent than free DCPIP in the ability to down-regulate pro-angiogenic and pro-inflammatory factors, VEGF, COX-2, IL-6 and IL-8, in cultured human colon cancer cells; (c) DCPIP and DCPIP NPs are similarly effective in inhibiting VEGF-induced angiogenesis *in ovo*; (d) PLGA NPs can serve as a useful vehicle for the delivery of DCPIP; and (e) DCPIP causes apoptosis of HCT116 cells at concentrations ≥ 6 μg/ml.

## Methods

### Reagents

All chemicals and cell culture reagents were purchased from Sigma Chemical Co (St. Louis, MO). Antibody to VEGF was obtained from Santa Cruz (Santa Cruz, CA). Antibodies to COX-2, caspase-3 and PARP were purchased from Cell Signaling (Danvers, MA); and antibodies to IL-6, IL-8 and β-actin were obtained from Abcam (Cambridge, MA). All antibodies used in this study were raised in rabbits. Fertilized leghorn chicken eggs used in the angiogenesis studies were purchased from CBT Farms (Federalsburg, MD).

### Cells

Human colon cancer HCT116 cells were obtained from the American Type Culture Collection (ATCC, Manassas, VA) and grown in Dulbecco's modified Eagle medium (DMEM) containing 10% heat-inactivated fetal bovine serum (FBS) and 1% penicillin-streptomycin (PS). The cell cultures were grown in a 5% CO_2_-atmosphere of a humidified incubator at 37°C.

### Synthesis and characterization of DCPIP-PLGA NPs

Poly(lactic-co-glycolic) acid nanoparticles (PLGA NPs) were synthesized using a double emulsion solvent evaporation technique [39]. Briefly, to 1 ml of 30 mg/ml PLGA in chloroform (CHCl_3_), 200 μl of 3 mg/ml DCPIP in water were added and vortexed. This primary emulsion was then transferred into 10 ml of 2% (w/v) polyvinyl alcohol (PVA), which acts as a surfactant, and the entire solution was sonicated on ice for 3 min using a probe sonicator (Misonix XL-2000, Newtown, CT). The organic solvent in the final solution was allowed to evaporate overnight with continuous stirring. DCPIP-containing PLGA NPs (referred to as DCPIP NPs, hereafter) were recovered by centrifugation at 20,000 × g for 20 min at 4°C. The pellet consisting of aggregated NPs was washed three times in water to remove any residual PVA and free, i.e., non-encapsulated, DCPIP. DCPIP NPs were then re-suspended in water, freeze-dried for 24 hr, and then stored at -20°C for future use. The amount of encapsulated DCPIP was quantified using HPLC and is presented as μg of DCPIP/mg of PLGA. This is referred to as the drug loading amount. Size, polydispersity index, and ζ-potential measurements of synthesized DCPIP NPs were determined by diffraction light scattering (DLS) utilizing a Zeta PALS instrument (Brookhaven Instruments, Holtsville, NY). The ζ-potential is the electric potential difference between the dispersion medium and the fixed layer of fluid attached to the dispersed nanoparticles. Surface morphology of the NPs was examined using a JOEL-JSM-880 scanning electron microscope (SEM). To determine the amount of DCPIP released from the NPs, DCPIP NPs were incubated in PBS pH 7.4 at 37°C on an orbital shaker. At pre-defined time points, aliquots were taken and centrifuged at 20,000 × g for 20 min. Supernatants were saved and later assayed using HPLC.

### Cell proliferation assay

The toxicity of DCPIP and DCPIP NPs on the proliferation of HCT116 cells was determined by using the XTT-based In Vitro Toxicology Assay kit (Sigma-Aldrich). Briefly, XTT also known as 2,3-bis[2-methoxy-4-nitro-5-sulfophenyl]-2H-tetrazolium-5-carboxyanilide inner salt is a yellow-colored salt, which when added to cell cultures is cleaved by metabolically active cells to form an orange-colored formazan dye. The amount of formazan dye is directly proportional to the number of living cells. In this study, HCT116 cells were seeded on 96-well plates at a density of 1 × 10^3 ^cells/well and allowed to adhere overnight. The cells were then treated with increasing concentrations of DCPIP or DCPIP NPs in DMEM with 10% FBS, and 1% P/S for 48 hr and incubated at 37°C. Proliferation activity was determined by treating the cells with XTT for 2 hr, i.e., XTT was added to treated and control untreated cells on the 46^th ^hr of treatment. The absorbance was then measured at 450 nm with reference wavelength of 690 nm using the Synergy HT plate reader (BioTek Instruments, Winooski, VT). The GI50 and LC50 were determined according to an established methodology [40,41]. We have used this methodology in previous studies reported elsewhere [42-44].

### Western blot analysis

HCT116 cells were exposed to increasing (2-fold) concentrations of DCPIP or DCPIP NPs for 48 hr. Whole cell lysates were collected in electrophoresis SDS sample-buffer and total cell protein concentration was determined using the Micro BCA Protein Assay kit (ThermoScientific, Waltham, MA). Lysate aliquots containing 35 μg total cell protein per aliquot were subjected to electrophoresis analysis on a 10% polyacrylamide/Tris-glycine/SDS gel. Proteins on the gel were subsequently transferred onto nitrocellulose membranes, which were subsequently incubated with antibodies to VEGF, COX-2, IL-6 IL-8, caspase-3 and PARP overnight at 4°C, and β-actin for 1 hr at room temperature. The membranes were developed using the SuperSignal West Pico Chemiluminescent Substrate (Pierce, Rockford, IL). Images of proteins were visualized using the Alpha Innotech HD2 (Alpha Innotech, San Leandro, CA).

### Chick chorioallantoic membrane assay of angiogenesis

The chick chorioallantoic membrane **(**CAM) assay is a standard assay for testing various agents for anti-angiogenic effectiveness [45]. The CAM assay used in this study was performed according to the modified version of Brooks et al [46]. Briefly, fertile leghorn chicken eggs were incubated at 37°C for 10 days turning them at regular intervals to achieve nearly complete vasculogenesis and blood vessel development progressing mostly through angiogenesis. On day 10, the eggs were candled and a small circular opening was made at the top of the eggs. DCPIP or DCPIP NPs were studied for anti-angiogenic ability in the absence or presence of 100 ng of human VEGF applied to 6-mm diameter paper discs and placed on the CAMs. The holes were covered with tape and the eggs were then incubated for 48 hr, and subsequently the CAMs were fixed and digitized images were recorded using a dissecting microscope (Amscope, model MD600). Data were analyzed using one-way ANOVA to compare the means of all groups. The Dunnett's multiple-comparison test was used to compare the treated versus the control groups, and unpaired two-tailed Student's *t*-test was used to compare treated groups to one another. A statistical difference was considered significant when *p *< 0.05. All analyses were performed with the aid of Prism 5.0 software (Graphpad Software, San Diego, CA).

### I*κ*B-luciferase degradation assay

Degradation of the NF-κB/IκB complex was detected by the Genetic Expression and Measurement (GEM™) assay using HCT116 cells stably transfected with PGL3-IκB firefly luciferase and referred to as HCT116/IκB-Luc cells. The methodology to generate HCT116/IκB-Luc cells and the principle of the GEM assay have been described [47]. Briefly, the principle is based on the ability of tumor necrosis factor-α (TNF-α) to induce degradation of IκB bound to NF-κB and thus generate active NF-κB, allowing the unbound NF-κB to translocate into the nucleus. The extent of the remaining intact IκB can be relatively quantified by monitoring the extent of a fluorochrome bound to IκB. In this study, HCT116/IκB-Luc cells were seeded on 96-well plates at a density of 3 × 10^4 ^cells/well and allowed to adhere overnight to the plastic substrate. The cells were then treated with increasing (2-fold) concentrations of DCPIP or DCPIP NPs in DMEM/10% FBS, and 1% P/S for 2 hr followed by treatment with TNF-α for 30 min at 37°C. After washing with PBS, cell lysis buffer was added and the absorbance of the lysates was then measured at 260 nm using the Synergy HT plate reader (BioTek Instruments, Winooski, VT). The results are reported as mean ± SEM. All measurements were performed in triplicate. Statistical differences between treatments were evaluated using Student's *t*-test and were considered significant when *p *< 0.05.

## Results

### Synthesis and characterization of DCPIP-containing PLGA NPs

**Figure 1:**
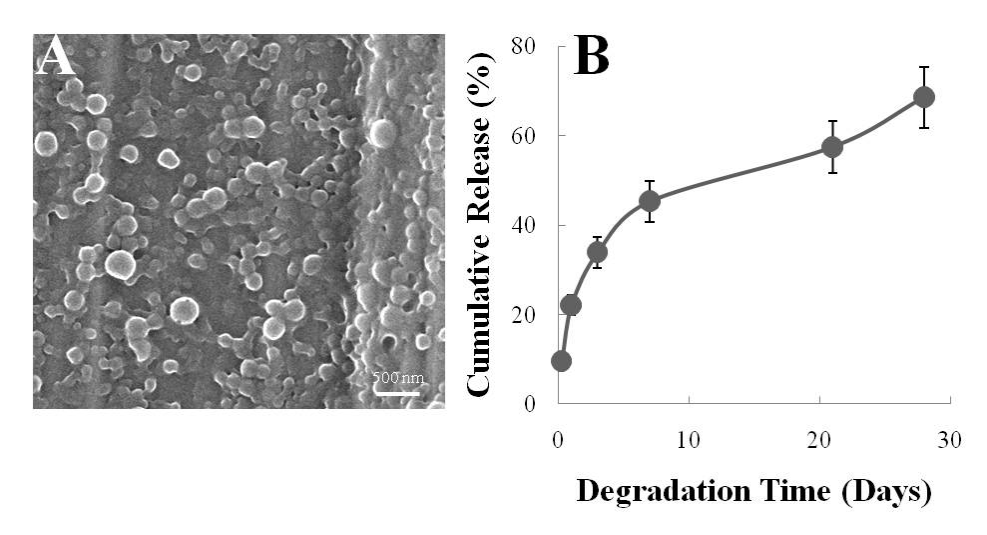
**Morphology and degradation of DCPIP NPs**. A. Morphology of DCPIP NPs as observed by scanning electron microscopy, and B. Kinetics of degradation of DCPIP NPs over a period of 28 days. The measurements are reported as mean ± SD (n = 3).

PLGA NPs were formulated and used to encapsulate DCPIP. There was no critical difference in size and ζ-potential between PLGA NPs, i.e. empty NPs, and DCPIP NPs, i.e. DCPIP-containing PLGA NPs (Table 1). The drug loading amount was calculated to be 7.45 μg DCPIP/mg of PLGA NP. The polydispersity index indicates that the NPs are monodisperse with nearly uniform size distribution. The PLGA NPs appear as spherically shaped particles, by scanning electron microscopy, consistent with published reports on PLGA NPs constructed by the double emulsion method (Figure 1A). Degradation of DCPIP NPs indicated an initial burst release by day 1, followed by a continuous release pattern that lasted until day 28 of the study (Figure 1B).

**Table 1:** Characteristics of PLGA NPs and DCPIP NPs

	*PLGA NPs*	*DCPIP NPs*
**Size (nm)**	174.4 ± 1.7	169.0 ± 0.6
**Polydispersity Index**	0.032 ± 0.011	0.073 ± 0.012
**ζ-Potential (mV)**	-37.68 ± 1.45	-44.21 ± 2.69
**Encapsulation Efficiency**	N/A	7.45 μg*

* 7.45 μg DCPIP per mg of PLGA NPs

### Cell proliferation assay

**Figure 2:**
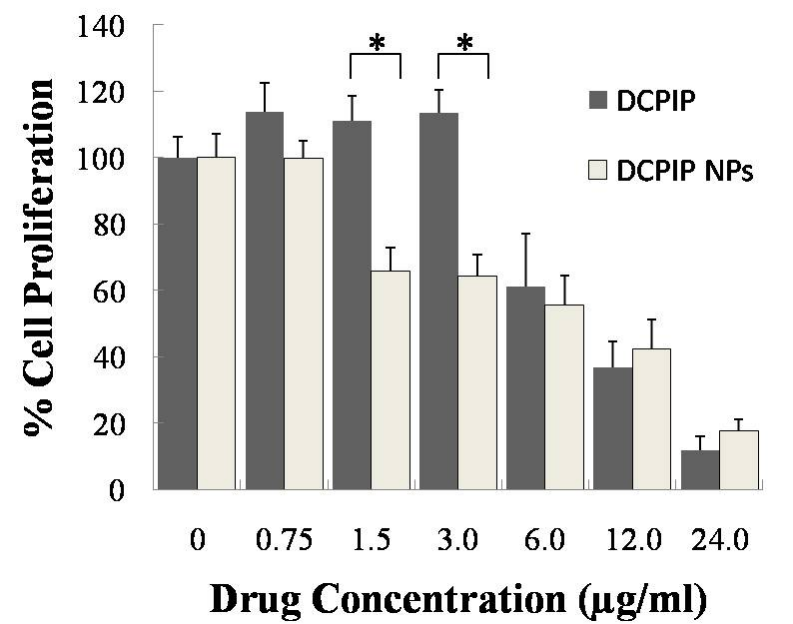
**Proliferation of HCT116 cells treated with DCPIP and DCPIP NPs**. Cell proliferation was determined by the XTT method. The concentrations of the free DCPIP and DCPIP NPs were calculated to be equal. Proliferation of untreated cells (0 μg) was taken as 100%. The measurements of the treated cells were normalized to the control measurement (100%). All measurements are reported as mean ± SD (n = 3). The asterisk (*) indicates *p *< 0.05.

The toxicity of free DCPIP and DCPIP NPs on HCT116 cells was determined by the XTT method for cell proliferation. The concentrations of DCPIP NPs treatments were calculated in such a way to include equivalent amounts as the DCPIP treatments. The cells were treated with various concentrations of free DCPIP and DCPIP NPs for 48 hr at 37°C. All measurements were normalized to the measurement of the control untreated cells which was considered to be 100%. Our initial observation was that treatment of the cells with DCPIP concentrations less than 6 μg/ml did not affect the cell proliferation activity, whereas, 1.5-6.0 μg/ml DCPIP NPs concentrations appeared to be moderately more effective than free DCPIP in inhibiting cell proliferation (Figure 2). However, higher concentrations of DCPIP appeared to have similar effectiveness as DCPIP NPs, This cell toxicity was particularly apparent in cells treated with 24 μg/ml of DCPIP and DCPIP NPs. The GI_50_, i.e. the concentration that inhibits the growth of 50% of the cells, and LC_50_, i.e. the lethal concentration that kills 50% of the cells, were determined to be about 9 μg/ml and 20.3 μg/ml, respectively, for DCPIP, and 2.4 μg/ml and 16.4 μg/ml, respectively, for DCPIP NPs. These concentrations indicate that the DCPIP NPs were more potent than the DCPIP alone for the same concentration.

### Western blot analysis

**Figure 3:**
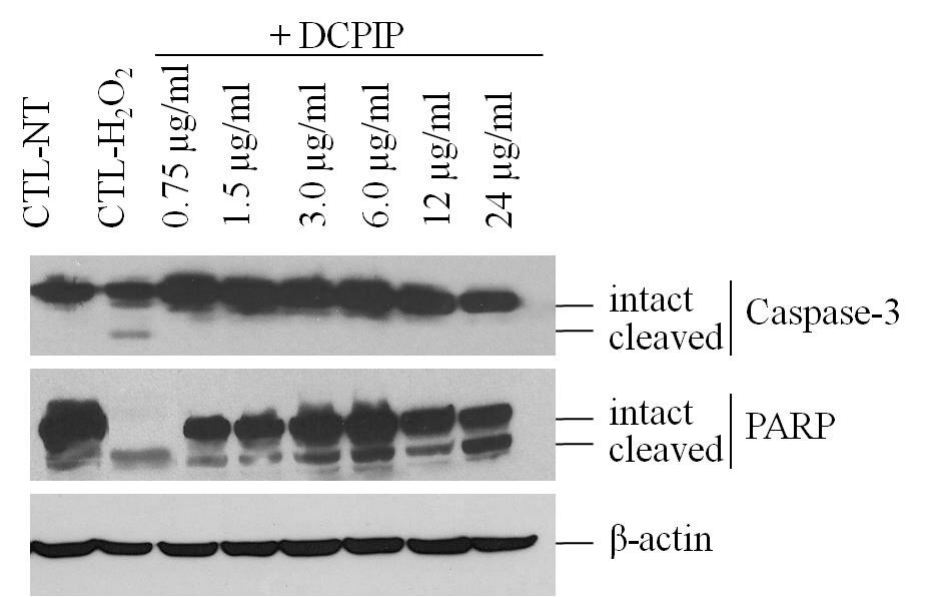
**Detection of cell death mechanism by Western blot**. HCT116 cells were treated with increasing concentrations of DCPIP (2-fold) for 48 hr, and then, aliquots of 35 μg of whole cell protein were subjected to analysis for detection of caspase-3, PARP and β-actin proteins. β-actin was used as a cell internal protein marker. The control groups included untreated cells (CTL-NT) and cells treated with 0.15 μg/ml H_2_O_2_.

To determine the mechanism of cell death caused by high concentrations of DCPIP (higher than 6 μg/ml), Western blot analysis was used to probe for caspase-3 and PARP. Hydrogen peroxide was used as a positive control as it has been shown to induce cell apoptosis [48]. There was a slight decrease in intact caspase-3 expression after treatment with 12 and 24 μg/ml DCPIP. However, no expression of cleaved caspase-3 was detected for all DCPIP treatments. Further, the expression of intact PARP appeared to decrease and was dependent on DCPIP dose (Figure 3). Concurrently, the amount of cleaved PARP appeared to increase. Degradation of caspase-3 and PARP was specific as indicated by the fact that the cell internal control, β-actin, remained intact.

**Figure 4:**
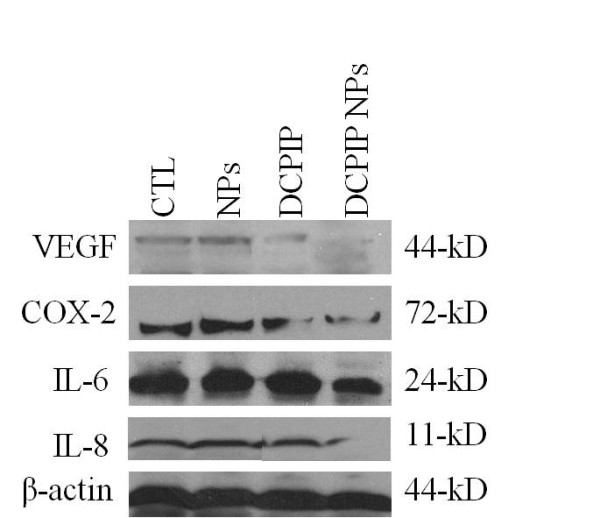
**Detection of angiogenesis- and inflammation-inducing factors by Western blot analysis**. HCT116 cells were treated with empty PLGA NPs (control), DCPIP, and DCPIP NPs for 48 hr, then, aliquots of 35 μg of whole cell protein were subjected to analysis for detection of VEGF, COX-2, IL-6, IL-8 and β-actin proteins. β-actin was used as a cell internal protein marker. An additional control included untreated (CTL) cells.

**Figure 5:**
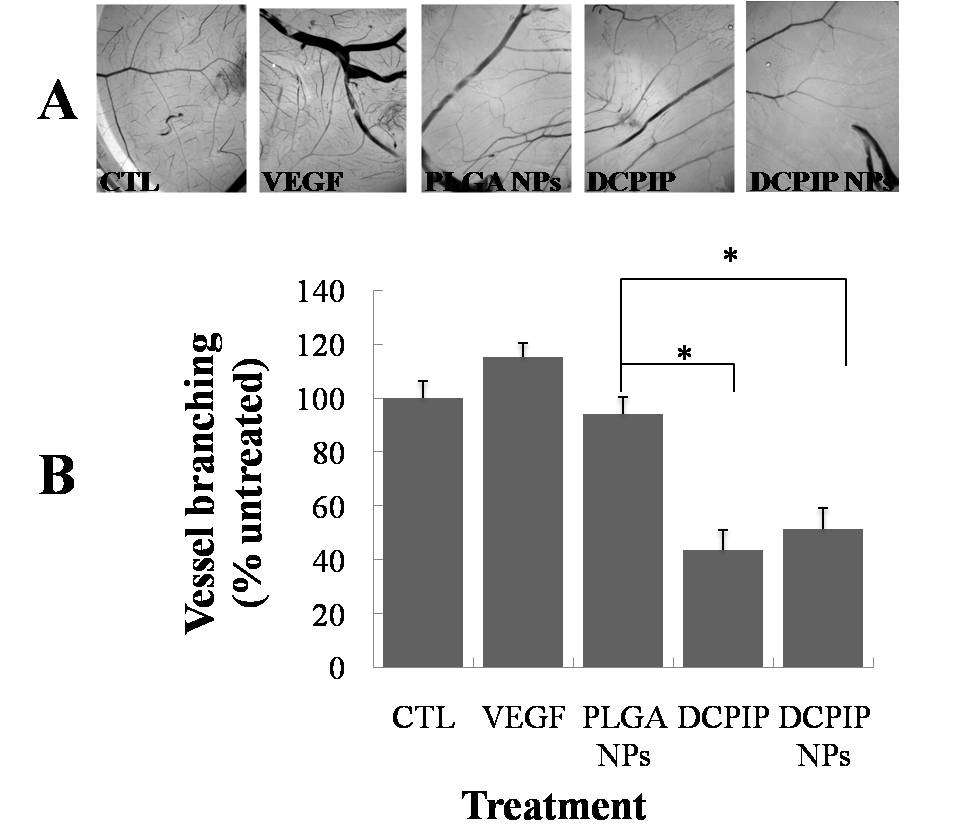
**Effect of DCPIP and DCPIP NPs on VEGF-induced angiogenesis in chicken eggs**. CAMs were treated with the reagents indicated in the figure for 48 hours as described in the Materials section (panel A). Visual quantitative results (angiogenesis index) for each experimental variable are also shown (panel B). The blood vessels, in randomly selected CAM fields of the eggs, were photographed and counted. The results from three eggs per treatment are reported as mean ± SD. The asterisk (*) indicates *p *< 0.05 as compared to empty PLGA NPs.

To determine the effect of DCPIP and DCPIP NPs on angiogenesis and inflammation, Western blot analysis was used to probe for the expression of angiogenic factor VEGF and inflammatory markers COX-2, IL-6 and IL-8. Both DCPIP and DCPIP NPs downregulated VEGF expression (Figure 4) consistent with the results obtained from the CAM assay (see below, and Figure 5). However, only DCPIP NPs significantly reduced the expressions of COX-2, IL-6 and IL-8 (Figure 4).

### Chick chorioallantoic membrane assay of angiogenesis

To study the effect of DCPIP and DCPIP NPs on vascularization, we used the CAM angiogenesis model (Figure 5A). The fertilized eggs were treated with 24 μg of either DCPIP or DCPIP NPs. Compared to both controls (control #1-no treatment, control #2-PLGA NPs), both DCPIP and DCPIP NPs significantly reduced the number of blood vessels after 48 hr treatment (Figure 5B).

### Degradation of the NF-κB/IκB complex monitored by the GEM assay

**Figure 6:**
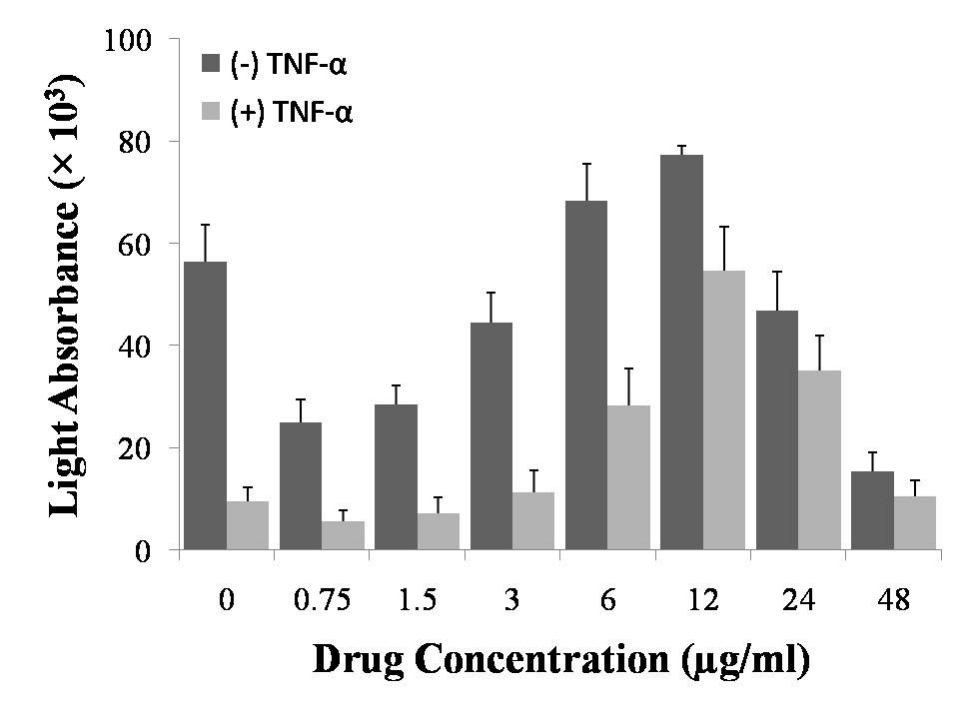
**Genetic Expression and Measurement (GEM) assay**. Detection of involvement of NF-κB pathway, in presence and absence of TNF-α, in HCT116-IκB-Luc cells treated with DCPIP for 2 hr. The measurements are reported as mean ± SD (n = 3).

To determine whether VEGF, COX-2, and IL-8 expressions are related to the NF-κB signaling pathway, in the HCT116 cells treated with DCPIP and DCPIP NPs, we used our novel GEM assay that was recently developed in our lab. For this reason, HCT116 cells were stably transfected with PGL3-IκB firefly luciferase (IκB-Luc) plasmid. The results have shown that DCPIP at concentrations of 6, 12 and 24 μg/ml confers the most protection for IκB (Figure 6). Treatments with DCPIP NPs did not show any significant difference (data not shown). This is probably because the treatments were given for 2 hr, not enough time for the NPs to degrade and release significant amounts of DCPIP.

## Discussion

In this report, we have demonstrated that (a) DCPIP enclosed in PLGA nanoparticles (DCPIP NPs) exhibits higher anti-proliferative activity than free DCPIP in cultured cancer HCT116 cells; (b) DCPIP NPs is similarly or more potent than free DCPIP in the ability to down-regulate pro-angiogenic and pro-inflammatory factors, VEGF, COX-2, IL-6 and IL-8, in cultured human colon cancer cells; (c) DCPIP and DCPIP NPs are similarly effective in inhibiting VEGF-induced angiogenesis *in ovo*; (d) PLGA NPs may serve as a useful vehicle for the delivery of DCPIP; and (e) DCPIP causes apoptosis of HCT116 cells at concentrations ≥ 6 μg/ml.

The redox dye DCPIP (2,6-dichlorophenolindophenol) has been previously used to demonstrate that complexes of enzymes are involved in transferring electrons to and from NAD(P)H in normal and neoplastic hepatocytes [26]. Moreover, DCPIP has demonstrated anticancer activity against human melanoma cells *in vitro *and *in vivo *[30]. In this context, we investigated the ability of DCPIP and nanoparticle-encapsulated DCPIP to affect the expression of factors/cytokines inducing or being associated with the processes of angiogenesis and inflammation. In general, nanoparticle-mediated delivery has been considered to enhance the bioavailability of an active component such as a drug, while limiting toxicity. Thus, nanoparticle delivery systems are promising tools for treatment of infectious diseases with vaccines [49,50], chemoprevention or treatment of cancer [51-54], initiation or inhibition of immune responses and angiogenesis [55-59], and treatment of various diseases [60-62].

We initially constructed and subsequently characterized PLGA nanoparticles containing DCPIP (DCPIP NPs). The DCPIP NPs, in PBS at 37°C, displayed a release profile, characteristic of an initial burst followed by a relatively constant release until day 28 of the study. The burst release is characteristic of hydrophilic drugs encapsulated inside polymeric nanoparticles. This burst release could be explained due to the fact that the hydrophilic drug has readily escaped or diffused into the aqueous medium under a concentration gradient. The relatively constant release that follows is primarily due to the hydrolysis of the ester bonds between the individual monomer which causes the degradation of the nanoparticles and hence the sustained release and bioavailability of DCPIP.

In the comparative efficiency studies between DCPIP and DCPIP NPs, we first considered to use equal amounts of free and encapsulated DCPIP. Thus, we calculated all the amounts of the encapsulated DCPIP after HPLC studies determined that the constructed DCPIP NPs contained approximately 7.45 μg DCPIP per mg of PLGA NPs (see Materials section, and Table 1). This way, for any amount of free DCPIP, we used equal amount of encapsulated DCPIP regardless of the amount of the PLGA moiety. We then, conducted pilot studies to select the most appropriate period of treatment of the HCT116 cells with DCPIP. In these studies, we initially treated the cells for various periods of time, ranging from 2 hr to 72 hr, with various concentrations of DCPIP NPs and the equivalent free DCPIP concentration. Free DCPIP elicited results as early as 2 hr of treatment and resulted in cell detachment from the substrate at treatment periods longer than 48 hr. Apparently, free DCPIP was toxic for the cells when the treatment periods were longer than 48 hr. However, DCPIP NPs treatments up to 24 hr did not exhibit any effect on the cells presumably because only a small amount of DCPIP was released from the DCPIP NPs during treatments for 2 hr to 24 hr, which is in agreement with the kinetics of DCPIP NPs degradation in PBS (see Figure 1). These findings were further confirmed by carrying out a standard cell proliferation (or growth inhibition) assay which indicated that a DCPIP NPs concentration, i.e., 2.4 μg/ml, was adequate to result in a GI_50 _caused by a higher free DCPIP concentration, i.e., 9 μg/ml. Therefore, taking into consideration these observations, we chose to treat the HCT116 cells with DCPIP and DCPIP NPs for 48 hr in the various studies despite the demonstration that much more DCPIP would be freed from DCPIP NPs for treatment periods longer that 48 hr.

Further, we determined the mechanism of cell death induced by high concentrations of DCPIP. The Western blot analysis of caspase-3 shows a slight decrease in expression of intact caspase-3 after treatments of 12 and 24 μg/ml DCPIP with no expression of cleaved caspase-3 for all DCPIP treatments (Figure 3). However, expression of intact and cleaved PARP was dose-dependent (Figure 3). This shows that DCPIP concentrations ≥ 6 μg/ml induce apoptosis of HCT116 cells probably through a mechanism that is totally or partially independent of caspase-3. We and others have previously reported the existence of caspase-dependent and caspase-independent mechanisms in cells treated with various reagents [30-32].

Once we established the experimental parameters of concentrations and periods of treatments of HCT116 cells with DCPIP and DCPIP NPs, we compared the effects of these two agents on the expression of the 44-kDa growth factor, VEGF, and the 72-kDa enzyme, COX-2, which have been associated with angiogenesis, carcinogenesis, and increased incidence of distant metastasis [10], and are robustly expressed in the human colon cancer HCT116 cell line. The results demonstrated that both free DCPIP and DCPIP NPs extensively downregulated VEGF, while no effect was observed in the expression of VEGF in control HCT116 cells treated with empty PLGA NPs alone. Moreover, the effect of DCPIP NPs was more extensive than the effect of free DCPIP on the downregulation as observed by direct visualization of the density of protein bands (Figure 4). Also, it should be noted that this effect of DCPIP NPs was in actuality more dramatic than the observed one since only a fraction of DCPIP was released from the DCPIP NPs in 48 hr. In this context, DCPIP NPs were similarly more effective in down-regulating COX-2 expression in the HCT116 cells. In conclusion, DCPIP NPs are more efficient than free DCPIP in the ability to disrupt sustained expression of endogenous VEGF and COX-2 in HCT116 and perhaps cells derived from other cancer types.

We further investigated whether DCPIP and/or DCPIP NPs can regulate the pro-inflammatory cytokines IL-6 and IL-8, which play crucial roles in the pathogenesis of various diverse diseases including oncogenesis, atherosclerosis, sepsis and rheumatoid arthritis [11,14-17], and acquisition of chemotherapeutic resistance in androgen-independent proliferation of prostate cancer cells [14], and may or may not produced following induction by the presence of VEGF [11-14]. Our studies of Western blot analysis demonstrated that control (empty NPs) and free DCPIP did not significantly affect the expression of the 24-kDa IL-6 in HCT116 cells. However, DCPIP NPs did down-regulate the expression of IL-6 (Figure 4). Further, DCPIP had no effect on the expression of the 11-kDa IL-8, whereas, the presence of DCPIP NPs in the cell culture resulted in nearly complete inhibition of IL-8 expression. These results suggest, but do not prove, that there might be an association between cellular mechanisms involving VEGF and IL-8.

Our studies above demonstrated that DCPIP and DCPIP NPs are able to downregulate the expression of the major angiogenesis factor, VEGF. Therefore, we utilized the CAM assay, which is an *in ovo *standard assay for testing various agents for anti-angiogenic effectiveness induced by VEGF [45]. Compared to three controls (CTL, negative control with no treatment; VEGF, positive control with VEGF treatment; and PLGA NPS, negative control treatment with empty NPs), the presence of both free DCPIP and DCPIP NPs resulted in significantly reduced number of blood vessels on the CAMs of the eggs after 48 hr incubation (Figure 5B). These results unequivocally demonstrated that both DCPIP and DCPIP NPs are very potent inhibitors of angiogenesis; however, we could not conclude whether treatment with DCPIP NPs was more effective than treatment with free DCPIP.

There is extensive experimental and clinical evidence to indicate that the nuclear transcription factor-κB (NF-κB) is activated during, and therefore links, the processes of angiogenesis, inflammation and carcinogenesis [21-24]. Therefore, we investigated whether the results described in this study as well as studies published by others on the anti-melanoma activity of DCPIP *in vitro *and *in vivo *[27] can correlate with activation of NF-κB. To determine this correlation, we utilized our novel Genetic Expression and Measurement (GEM) assay which indicates whether an agent can block the ability of TNF-α to induce degradation of IκB bound to NF-κB, a complex that is located in the cell cytoplasm, and therefore prevent generation of active NF-κB and its translocation into the cell nucleus [47]. The GEM assay is described more extensively in the Methods section. Because degradation of the NF-κB/IκB complex is an early cellular event, treatment with DCPIP was only for 2 hr. The results indicated that the NF-κB/IκB complex was protected by the TNF-α induced degradation in the presence of 6-24 μg/ml DCPIP, with the highest protection conferred at a concentration of 12 μg/ml DCPIP (Figure 6).

## Conclusion

Our findings indicate that DCPIP may have potential therapeutic benefits against cancer and other diseases because of its anti-inflammatory and anti-angiogenic properties. Delivery of DCPIP using PLGA nanoparticles has shown to be equally or more effective than free DCPIP *in vitro *and *in ovo*. DCPIP concentrations ≥ 6 μg/ml induce HCT116 cell death through apoptosis. We have currently initiated an extensive investigation to optimize the effect of DCPIP NPs as compared to free DCPIP and determine the anticancer ability of DCPIP and DCPIP NPs in immunosuppressant mice carrying established human HCT116 tumors as well as other tumors of diverse origin.

## Competing interests

The authors declare that they have no competing interests.

## Authors' contributions

FGM participated in the design of all the experiments and performance of nanoparticle synthesis and characterization, XTT cell proliferation, Western blot, CAM assay and GEM assay experiments, assisted in data analysis and interpretation and in writing and revising the manuscript. SP and JG carried out the Western blot experiments. CH and SA assisted in the design planning of all experiments. PP participated in the design of XTT, CAM and Western experiments, assisted in data analysis and interpretation and in writing and revising the manuscript. RPR assisted in the design of all experiments and in writing and revising the manuscript. All authors have read and approved the final manuscript.
